# Perceptions of the Use of Mobile Apps to Assess Sleep-Dependent Memory in Older Adults With Subjective and Objective Cognitive Impairment: Focus Group Approach

**DOI:** 10.2196/68147

**Published:** 2025-04-28

**Authors:** Aaron Lam, Simone Simonetti, Angela D'Rozario, David Ireland, DanaKai Bradford, Jurgen Fripp, Sharon L Naismith

**Affiliations:** 1 Healthy Brain Ageing Program, The Brain and Mind Centre, The University of Sydney, 96 Mallett Street, Camperdown, 2050, Australia, 61 435032636; 2 School of Psychology, The University of Sydney, Camperdown, Australia; 3 Woolcock Institute of Medical Research, Macquarie University, Macquarie Park, Australia; 4 Australian eHealth Research Centre, Commonwealth Scientific and Industrial Research Organisation, Herston, Australia

**Keywords:** aging, mild cognitive impairment, subjective cognitive impairment, digital health, cognition, neuropsychology, sleep

## Abstract

**Background:**

Sleep-dependent memory (SDM) is the phenomenon where newly obtained memory traces are consolidated from short-term memory stores to long-term memory, underpinning memory for daily life. Administering SDM tasks presents considerable challenges, particularly for older adults with memory concerns, due to the need for sleep laboratories and research staff being present to administer the task. In response, we have developed a prototype mobile app aimed at automating the data collection process.

**Objective:**

This study investigates the perspectives of older adults, with subjective or objective cognitive impairment, regarding barriers and facilitators to using a new mobile app for at-home assessment of SDM.

**Methods:**

In total, 11 participants aged 50 years and older were recruited from the Healthy Brain Ageing memory clinic, a specialized research memory clinic that focuses on the assessment and early intervention of cognitive decline. Two focus groups were conducted and thematically analyzed using NVivo (version 13; Lumivero).

**Results:**

On average, participants were aged 68.5 (SD 5.1) years, and 4/11 were male. Eight participants had subjective cognitive impairment, and 3 participants had mild cognitive (objective) impairment. Two main themes emerged from the focus groups, shedding light on participants’ use of mobile phones and the challenges and facilitators associated with transitioning from traditional laboratory-based assessments to home assessments. These challenges include maintaining accurate data, engaging with humans versus robots, and ensuring accessibility and task compliance. Additionally, potential solutions to these challenges were identified.

**Conclusions:**

Our findings underscore the importance of app flexibility in accommodating diverse user needs and preferences as well as in overcoming barriers. While some individuals required high-level assistance, others expressed the ability to navigate the app independently or with minimal support. In conclusion, older adults provided valuable insights into the app modifications, user needs, and accessibility requirements enabling home-based SDM assessment.

## Introduction

There are over 55 million people living with dementia [1]. Dementia is an umbrella term for a diverse number of substantial cognitive impairments that significantly interfere with an individual’s daily living. The most common form of dementia is Alzheimer disease, in which memory impairment is a critical early feature [2] that is intricately linked to diminished well-being and quality of life [3]. Some facets of memory can be assessed by standardized neuropsychological tests that examine a person’s ability to recall information presented to them after a short delay (eg, 20‐30 minutes). An aspect of memory that cannot be assessed during a standardized neuropsychological test is sleep-dependent memory (SDM). This is a process by which memories encoded during the day are consolidated and strengthened during sleep [4]. This mechanism greatly influences overall memory capabilities by bolstering long-term memory storage, integration, and retrieval.

To date, most studies have explored SDM in younger adults [5]. Thus, tasks are tailored to suit the cognitive capacities of younger individuals and often involve memorizing many items. This poses significant challenges for older adults, particularly those with cognitive impairments, leading to a notable research gap in this demographic. Given this gap and the importance of SDM in memory for daily life, our team developed an SDM task [6] tailored explicitly for older adults with cognitive concerns and mild cognitive impairment (MCI). MCI refers to a transitional state between normal aging and dementia, where cognitive impairment is apparent, but daily functioning is largely intact. Our prior work demonstrated that individuals with multiple-domain MCI had significantly more compromised SDM than healthy controls and those with single-domain MCI [6]. In this study, poorer SDM was linked to having greater sleep apnea severity for older adults without MCI. In contrast, for those with MCI, poorer performance was associated with decreased sleep spindle duration and smaller hippocampal subfield size. As various age-related sleep changes occur both naturally [7] and with neurodegenerative diseases [8,9], it is crucial to understand how these alterations impact SDM. Notably, preliminary evidence suggests that SDM may be improved through sleep apnea management [10], transcranial electrical stimulation [11], and acoustic stimulation [12]. These findings underscore the importance of identifying clinical correlates and predictors of poor SDM performance to inform targeted interventions.

Unfortunately, our understanding of SDM is limited to small sample case-control studies, and it is not routinely assessed in clinical evaluations. This may be partly attributable to the type of task used and the setting within which most research has been conducted. In addition to being predominantly in younger healthy samples, studies to date have largely been conducted in sleep laboratories where participants are asked to do memory testing before and after sleep [13]. This requires participants to sleep in an unfamiliar environment, meaning their sleep that night may not reflect their usual sleep [14], and they are often asked to learn significant amounts of information before sleep using tasks that are not feasible or suitable for clinical settings. Another barrier is the high demand for staff to conduct these studies (Naismith, personal communication, 2024). Staff must be present to administer SDM tasks before sleep (often within 3 hours before sleep) and after sleep (often after 1-hour postwaking) and to stay overnight to monitor the participant. Together, the requirement for sleep laboratories and high staff demand result in high costs associated with conducting SDM tasks. These barriers could be addressed by adapting SDM tasks for older clinical samples and delivering the tasks in the home environment.

The adoption of digital health technology in older adults has been increasing [15], including by individuals with subjective concerns about their cognition and those with MCI [16]. However, when considering new app-based approaches, barriers and facilitators that influence adoption and effective use must be identified [17]. A prior systematic review showed that barriers preventing the use of digital technology in older adults (including those with cognitive impairment) can include motor, sensory, cognitive, lack of familiarity, and device-specific challenges, though these factors can be mitigated through design modifications [18]. In contrast, digital health technology adoption can be facilitated by the perceived usefulness of the app, pre-existing knowledge, and ease of use. For SDM tasks, additional considerations are required, given that they are optimally tested under strict timings (ie, before and after sleep) and may require participants’ sustained attention and interaction with a mobile app for extended periods (eg, potentially up to an hour).

This study sought to explore the perspectives of older adults with subjective or objective cognitive impairment regarding barriers to and facilitators of an at-home assessment of SDM using a new “chatbot” (mobile app), approach to assessment, named “Sleep Memories.” A chatbot is designed to simulate human-like conversation with users through text or voice interactions and, in our scenario, to deliver a memory task and record a participant’s responses. Previous work has shown the use of chatbots in various health populations, including chronic pain populations to deliver pain education [19], and monitoring of chemotherapy outcomes in older adults with cancer [20]. The objective was to use co-design methodologies to assess an initial app prototype, which could be incorporated into a revised version suitable for clinical testing. We were specifically interested in potential barriers to and facilitators of use.

## Methods

### Digitalization of the SDM Task

**Figure 1.**
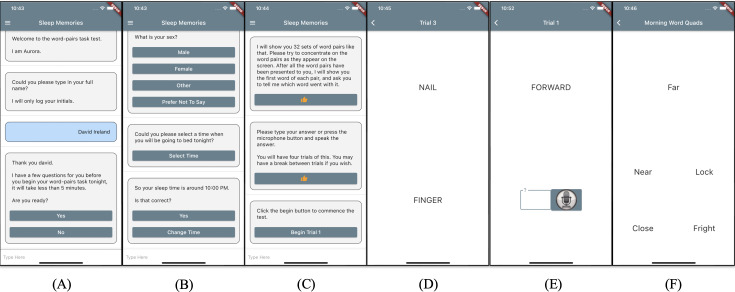
Screen captures of the Sleep Memories app. (**A**) Aurora (chatbot) introducing themselves to the participant. (**B**) Aurora asking demographic and habitual sleep pattern questions. (**C**) Aurora giving instructions for the word-pairs task. (**D**) Learning component of the word-pairs task being delivered by the app (**E**) Recall component of the word-pairs task being delivered by the app. (**F**) Multiple-choice testing component of the word-pairs task being delivered by the app.

The SDM task, as described in Lam et al [6], was initially adapted to be suited for an app-based platform (Figure 1). This task is a verbal memory task that comprises 32 word pairs, half of which are semantically related and the other half unrelated. In step 1, participants are presented with all 32 pairs. In step 2, individual words are presented, for which participants are required to identify the corresponding pair word by speech or text. As described in the initial study [6], participants performed this task 4 times in the evening within 3 hours of bedtime to facilitate learning. In the morning, participants completed the recall component (step 2) and a multiple-choice test, comprising an individual word and 4 possible answers (1 correct corresponding pair word and 3 incorrect pair words), approximately 1 hour after waking. This task was digitalized for use in a mobile app, using a chatbot for automatic delivery and data collection. To humanize the chatbot for prototype testing, we named it “Aurora.” The Sleep Memories app’s development, in-house testing, and evaluation are detailed in Ireland et al [21].

Sleep Memories was designed to collect information about a participant’s habitual sleep patterns, through a short survey on opening the app, and deliver the SDM task within 3 hours of the participant’s regular sleep time. After the participants respond to the questions about their sleep, the app provides a trial run, enabling participants to become comfortable with its functionalities. In line with Lam et al [6], each word pair is presented to the participants for 10 seconds. For the recall component, the app offers a 10-second window, allowing participants to either type or vocally record their answers, ensuring a user-friendly response period. In the morning, participants are prompted to confirm their bedtime and wake-up time, which is followed by recall and multiple-choice testing to assess their memory retention.

Qualitative methods were selected for this study, as they are ideally suited to elicit participants’ perspectives of Sleep Memories and their experiences with current digital health technologies relative to other methodologies. An interpretative philosophical perspective [22] was selected, as this method allowed us to situate the participants’ perspectives within their broader life contexts and allowed for flexibility and adaptability regarding the emergent themes and meanings arising from the data.

### Participant Recruitment

Participants were recruited via convenience sampling from the Healthy Brain Ageing (HBA) memory clinic. As described previously [23], the HBA clinic is a specialized memory clinic that accepts referrals from general practitioners and medical specialists and provides comprehensive neuropsychological, medical, and mood assessments for older adults aged >50 years with recent subjective cognitive or mood decline. Exclusion criteria are a Mini-Mental State Examination score<20 [24], intellectual disability, insufficient English proficiency for standardized neuropsychological assessment, history of nonaffective psychiatric disorder (eg, schizophrenia), history of stroke, history of head injury (loss of consciousness>30 minutes) or other neurological disorder (eg, epilepsy), or current substance dependence or abuse. Basic demographic (eg, age, sex, and years of education) and clinical data (eg, Mini-Mental State Examination score and MCI status) collected at the HBA clinic are reported here for descriptive purposes. MCI status was categorized into 3 distinct groups: subjective cognitive impairment (absence of objective cognitive impairment), nonamnestic MCI (objective cognitive impairment in nonmemory domains), and amnestic MCI (defined by objective cognitive impairment in memory domain). Participants were invited to participate in the study via email or phone call. Interested participants were provided with a participant information statement identifying the research team, funding, research aims, and risks and explaining confidentiality, consent, and withdrawal. Once informed consent was obtained, participants were invited to attend 1 of 2 digital focus groups via Zoom (Zoom Video Communications).

### Data Collection

Before the focus groups, participants were required to complete a digital survey consisting of an Abbreviated version of the previously validated Mobile Device Proficiency Questionnaire (AMDPQ) [25]. In general, the abbreviated version of the questionnaire captured 7 items on basic proficiency in mobile phone use (eg, charging the phone and typing with the keyboard), 8 items on proficiency in communication (eg, sending emails and messaging), 3 items on data and file storage (eg, transferring files from mobile to computer), 8 items on internet use, and 6 items on troubleshooting and software management (eg, updating and deleting apps). Higher scores on each item indicated greater proficiency in each aspect.

A semistructured focus group guide was developed in collaboration with AL (male, program manager), Peta Mills (female, clinical project officer, PhD), SS (female, postdoctoral research associate), and SLN (female, professor and clinical neuropsychologist with extensive experience in focus groups, workgroups, interviews, and working parties for a variety of purposes such as to develop guidelines or models). The focus groups and questions were structured around three core themes (see Multimedia Appendix 1 for the question guidelines used):

1. Current use of mobile phone apps: informed by prior literature [17,18], this theme focuses on participants’ current use of mobile phone apps, particularly for health and sleep-related functions. The theme includes use habits, types of health apps used, and experience with speech recognition features on mobile devices. Related questions were designed to assess participants’ familiarity with mobile phone technology, health-related apps, as well as their interactions and experiences with speech recognition features.
2. Interest in completing cognitive tasks through a mobile app: informed by previous studies [18,26], this theme was selected to explore participants’ willingness and comfort in using a mobile app for memory assessments. This theme includes the potential benefits and challenges of this approach compared to traditional methods. The aim is to understand the acceptability, motivation, practicality, and barriers or facilitators of adopting mobile apps for cognitive assessments.
3. Feedback on the proposed mobile app: this theme seeks to gather participants’ perspectives on various aspects of the proposed mobile app, including the clarity of instructions and tasks, visual appeal, potential distractions, and privacy concerns. Participants were also invited to provide suggestions for enhancing the usability and engagement of the mobile app. The questions specifically targeted feedback on the app’s features, such as the chatbot and task length or design, and were developed based on internal testing of the mobile app.

A Microsoft PowerPoint presentation (Multimedia Appendix 2) was used to guide participants through the focus group material. The presentation introduced the concept and significance of SDM, highlighting its potential impairment in aging and cognitive decline and its understudied nature due to current limitations in traditional data collection methods (ie, in a sleep laboratory setting). We showed how our mobile app may be a novel solution to these barriers to facilitate home-based assessments. The presentation also illustrated how participants would interact with Aurora, including the delivery of the word-pairs task, in which the process and structure of the SDM assessment involving learning and recall word pairs were explained.

Data were collected via 2 focus groups between October and November 2022. Each focus group was facilitated by separate researchers: SS, a postdoctoral research associate with 10 years of applied research experience and 4 years of qualitative research experience (interviews and focus groups), and Peta Mills, a clinical project officer with 11 years of applied research experience and 18 months of qualitative research experience (focus groups). At the time of the focus groups, both facilitators were not involved in this research project in any capacity and thus provided an impartial perspective. AL and DI (male, software engineer) attended the focus group sessions to answer participants’ logistical and technical questions. Focus groups lasted 60‐90 minutes and were audio-recorded, transcribed, and de-identified.

### Ethical Considerations

This study was approved by The University of Sydney Human Ethics Committee (2022/HE000563) and conducted in accordance with the World Medical Association Declaration of Helsinki. Written informed consent was obtained from all participants prior to any study procedures and given the option to opt out of the study at any given time. Participants were given a A$20 (US $12.75) gift voucher as compensation for their time. All data collected were deidentified to ensure participant privacy and confidentiality.

### Data Analysis

For clinical and survey data, frequency and descriptive statistics were computed using SPSS Statistics (version 24.0; IBM Corp). Focus group transcripts were analyzed thematically in NVivo (version 13; Lumivero), a qualitative data analysis computer software package, using the techniques described by Braun and Clarke [27]. The thematic analysis approach described by Braun and Clarke [27] is a systematic, iterative, and inductive process comprising 6 phases. Initially, researchers immerse themselves in the data to discern patterns and potential coding schemes. Subsequently, they generate initial codes to categorize salient features systematically. These codes are then organized into potential themes. Themes undergo rigorous validation to ensure coherence and alignment with the dataset. Once themes are finalized, they are defined, and any hierarchical relationships are identified. Finally, thematic insights are woven into a narrative to address relevant research questions.

As we had identified our 3 core themes during the development of the focus group guide, our thematic analysis was focused on a deeper exploration of these themes. Following Braun and Clarke [27] phases, AL and SS became familiar with the data via repeated reading and individually created initial codes for all transcripts by noting evocative phrases, ideas, and perceptions. Codes were then organized into meaningful groups and sorted into subthemes. Where there was disagreement in coding or theme development, the codes or themes were discussed and refined. To ensure the study conformed to best practice guidelines, we used the COREQ (Consolidated Criteria for Reporting Qualitative Research) checklist and met 27 of 32 items [28].

## Results

### Participants

In total, 19 people from the HBA memory clinic were invited to participate in the focus groups, and 11 of these agreed to participate. Their demographics and clinical information are reported in Table 1. Briefly, the sample on average was aged 68.5 (SD 5.1) years and predominately female (n=7, 64%). The sample also comprised 8 subjective cognitive impairment, 2 nonamnestic MCI, and 1 amnestic MCI. Of these participants, 10 completed the AMDPQ, with 1 participant declined, as they did not have sufficient proficiency to access the digital questionnaire. On average, participants demonstrated relatively high mobile proficiency (see Multimedia Appendix 3 for a full breakdown of participant responses to each individual item).

**Table 1.** Participant demographic and clinical information.

	Values
Age (years), mean (SD)	68.5 (5.1)
Sex (male), n (%)	4 (36)
Education (years), mean (SD)	15.1 (2.9)
MMSE^a^ score (out of 30), mean (SD)	29.2 (0.9)
Subjective cognitive impairment, n (%)	8 (73)
Nonamnestic mild cognitive impairment, n (%)	2 (18)
Amnestic mild cognitive impairment, n (%)	1 (9)
AMDPQ^b^ total score (out of 25), mean (SD)	20.5 (4.6)
AMDPQ—mobile device basic subscale (out of 5), mean (SD)	4.4 (0.6)
AMDPQ—communication subscale (out of 5), mean (SD)	4.0 (1.0)
AMDPQ—data and file storage subscale (out of 5), mean (SD)	3.7 (1.3)
AMDPQ—internet subscale (out of 5), mean (SD)	4.2 (1.1)
AMDPQ—troubleshooting and software management subscale (out of 5), mean (SD)	4.1 (1.1)

a MMSE: Mini-Mental State Examination.

b AMDPQ: Abbreviated Mobile Device Proficiency Questionnaire.

### Thematic Analysis

#### Overview

The thematic analysis revealed two broad themes relevant to the 3 core focus group themes: (1) context of mobile phone use and (2) shifting from traditional laboratory-based assessments to home assessments. The latter theme should be considered within the framework of the former theme, as shown in Textbox 1. That is, theme 2 considers the challenges and facilitators identified by participants when shifting from laboratory settings to home environments within 2 specific contexts: (1a) participants’ existing familiarity and proficiency with mobile phones and apps and (1b) the prevalent stereotypes and preconceived notions concerning aging and technology.

Textbox 1. **Breakdown of the overarching themes and subthemes identified in our study by 2 primary domains: “context of mobile phone use” and “shifting from traditional laboratory-based assessments to home assessments.”**

**Context of mobile phone use**

- Current phone use
- Aging

**Shifting from traditional laboratory-based assessments to home assessments**

- Accuracy of data
- Human versus robot
- Accessibility
- Compliance

#### Context of Mobile Phone Use

##### Current Phone Use

Participants were asked to share their mobile phone use patterns and specify the types of apps they used. Across participants, mobile phone use varied substantially from the use of basic phone functions, such as phone calls, text messaging, and camera functions, to more complex use involving apps, such as emails, social media, search engines, maps, news, weather, and music. Although minimally used, health and sleep apps were accessed for relaxation and to monitor sleep, heart rate, and physical activity. One participant felt highly proficient in phone use and that the phone was integral to the organization of their day-to-day life: “I would be lost without my mobile phone.” Speech-to-text functions were used as needed, particularly when driving and composing text messages, using navigation maps, or playing music. However, it is worth noting that not all participants chose to use this feature.

##### Aging

Individuals who reported limited phone use perceived their age as a barrier to gaining proficiency with new technologies.

I don’t think I am alone in this, but it is a real problem when you’re a bit older trying to pick up the technology that seems to be intuitive to other people.

Participants expressed a sense of not keeping pace with technology, which stemmed from their preference for relying on what they were familiar with or their self-perception as old-fashioned. Even when participants successfully used a new technology, they felt the need to use it continuously to avoid losing the knowledge of how to operate it effectively.

There is so much learning involved and if you don’t use these things more than once in 6 months it’s gone.

Irrespective of the reasons for their limited access to, and use of, technology, a prevailing sentiment among these individuals was a sense of missing out.

I have a Samsung phone, one of the latest, and I know there are lots of lovely things for me and I’m not using them. I would use them if they were set up.

Younger adults were viewed as having an innate familiarity with and competence in using digital tools and, thus, were flagged as potential facilitators of accessing and using technology. Subsequently, participants who did not have a younger adult available to facilitate their use of new technologies felt at a disadvantage. These observations reinforced the prevailing belief that older generations faced a variety of disadvantages with technology.

The insights gathered suggest a spectrum of technology assistance needs among participants. Some individuals may require comprehensive setup support, including step-by-step instructions and human interaction throughout their tech journey, while others might only require minimal assistance. This underscores the importance of adopting 1 of 2 design approaches for apps: either a straightforward and user-friendly design that prioritizes ease of use or, in the case of complex apps, the provision of substantial human support.

These findings carry significant implications for the design of apps intended for home use as opposed to controlled laboratory settings. They serve as a foundation for understanding the crucial factors and context that should inform the app design process when moving apps from the laboratory to the home.

### Shifting From Traditional Laboratory-Based Assessments to Home Assessments

#### Overview

There were several considerations for moving the SDM task from laboratory-based assessments to home assessments raised during focus groups. Participants identified facilitators as well as challenges and proposed potential solutions to overcome these obstacles (Table 2).

**Table 2.** Moving sleep-dependent memory from laboratory to home: challenges, solutions, and changes.

Challenge	Potential solution	App updates
Unexpected events during task completion (eg, phone ringing and someone knocking on the door).	Function to pause and recommence task.	The task was not implemented with a pause option because it could impact the results. Instead, Aurora advised participants to find a quiet, distraction-free place at the start of the task. If they could not finish because of unforeseen circumstances, they were offered another chance on a different night.
Difficulty with setting up apps and need help with troubleshooting.	Set up the app for participants and having someone to contact.	Through email, participants can reach a contact person who will provide technical support.
Participants did not want to enter identifying information (eg, names or date of birth).	Using a participant ID rather than identifying information.	We have given the participants an option to enter their participant ID. We now only collect participant initials and not name or date of birth.
Participants flagged that sometimes they ignore push notifications on their phones in the evening.	Option for the phone to ring or a text message.	The app now sends additional notifications to remind the participants to complete the task. Furthermore, the phone now sends short audio notification reminders to participants.

#### Accuracy of Data

Participants generally regarded the home environment as conducive to achieving higher accuracy levels relative to the laboratory environment, which was described as “ghastly” and distressing. Indeed, because of this perception, participants questioned the reliability of sleep laboratories, primarily because of the unconventional and unfamiliar sleep conditions they imposed. These doubts extended to the execution of the SDM task in the laboratory. Participants posited that conducting the SDM task in the laboratory might cause reduced accuracy compared to conducting the same tasks within the comfort of their homes, where they felt more relaxed as they did not have a researcher watching them and where they could expect a more restful night’s sleep. Similarly, there was a recognition that the peculiar sleep laboratory environment might introduce sleep disturbance that may disrupt SDM processes. Therefore, the results of the SDM task completed in a laboratory may not truly reflect an individual’s performance. To further illustrate these insights, one participant shared their personal experience with a sleep laboratory:

I recently, did a sleep study. It’s just not what I consider to be an indicator of anything that’s going on. I had a two-and-a-half-hour sleep if that through the whole night strapped into all these other things as well ... So definitely at home I think you’ll get more relaxed, and you get a true picture of what’s going on.

Conversely, having a researcher present and demanding attention could lead to better performance.

In the clinic, I was being put on the spot ... “we’re not just being casual here, we’re doing a trial and I’m seeking your recall results, so you need to pay attention.” So being in the clinic I was really on purpose for fear of failure ... At home I’d be far more relaxed and maybe that’s a good thing too.

In the laboratory setting, participants interact with the SDM task by saying their responses aloud. When considering the at-home mobile app version of the SDM task, participants were given a choice between typing their responses into an open text field or vocalizing their answers. Preferences varied, as participants felt that while using verbal responses may be more convenient than typing, the accuracy of verbal responses could be reduced. Nonetheless, being able to choose to use a text or a verbal response resonated positively with participants.

#### Human Versus Robot

During thematic analysis, some initial disagreements arose among the 2 scorers regarding the classification of human versus robot. AL suggested that the theme “human versus robot” may encompass accessibility as well, while SS suggested creating a separate theme for “accessibility.” To resolve this, the team engaged in iterative discussions, referring back to the original transcripts to ensure alignment with participants’ narratives. Through this process, a consensus was reached to have “human versus robot” and “accessibility” as 2 separate themes.

The facilitators explained to participants that they would primarily interact with Aurora, a chatbot designed to guide them through the SDM task via written text or verbal instructions. Participants overwhelmingly agreed that any kind of computerized intelligence should be as humanlike and natural as possible, suggesting that a computerized voice might be difficult to engage with.

I found it very easy to do it in the lab and the interaction with [researcher name] it was simple to do and it was a softer voice and it is relaxed and it is encouraging. All that subliminal processing of getting the task done.

Relying solely on a computerized and automated system without human support raised several challenges, and the lack of human interaction was primarily viewed as a drawback. Indeed, participants flagged the importance of human support, both during setup and for ongoing assistance. Participants also identified that the app may struggle to accommodate diverse accents or may lack the mechanism to clarify potential response errors, tasks that were easily accomplished by a human. To assist with these challenges, participants recommended that the app should be capable of detecting potential errors and should offer corrections or alternative suggestions when necessary.

Additionally, participants expressed apprehension around the security of data, given personal information was to be entered directly into the app. There was a hesitation to provide personal information, such as names or dates of birth. Instead, participants proposed using participant IDs only for entry into the app and that other information, such as date of birth, is gathered by the researcher in another way and is linked externally to this ID. However, other participants displayed indifference to this issue, expressing a belief that personal information is already widely accessible: “Everyone knows everything about us anyway.”

#### Accessibility

Participants identified 2 primary barriers that could impact the app’s accessibility. These were physical and economic barriers. Several participants expressed concerns regarding age-related vision problems, which can hinder their ability to read or view small text. Participants proposed using larger screens, like tablets, and increasing font sizes might address this barrier. Participant responses from the AMDPQ suggested that certain participants exhibited greater proficiency when using personal computers or tablets than smartphones, indicating the potential value of designing apps with flexibility and accessibility across different platforms.

I do I get a bit irritable with the smallest of the screen prefer to do things on the laptop.

Another physical barrier that was discussed was physical dexterity, which refers to fine motor skills and the ability to move their fingers. Age-related factors, such as tremors and arthritis, may be challenging for older adults to respond to Aurora or complete the SDM task.

The dexterity on the fingers may be important on how well someone clicks on the screen ... I might have trouble because I haven’t done it on a phone and only do it on a PC.

One participant raised concerns about whether the app required high bandwidth to use. High bandwidth apps can increase difficulty in accessing and using mobile apps as well as increase costs associated with using the app. Therefore, it is important to optimize apps for low bandwidth.

Is your application bandwidth heavy that it’s just going to slow things down for those of us who actually don’t have a proper wireless connection or a slow data connection and it’s just going to make it after a while just unusable nothing.

Participants noted that the initial setup phase of new technologies (eg, installing and setting up of apps) was either too challenging or time-consuming. Participants recognized that they required assistance or guidance to set up, navigate, and use technology effectively. This support could encompass various aspects, such as learning to use new devices, understanding complex software apps, or troubleshooting technical issues. However, they found that, in general, such support was lacking or insufficient. The consensus among participants strongly supports the idea that offering setup assistance, particularly during the initial stages of technology adoption, would be immensely beneficial. This will help familiarize participants with the mobile app, save time, and alleviate stress for older adults. Any kind of automation was also supported to avoid repeated data entry:

I don’t need to put that same information in every time that I’m doing this test because this would be 5‐10 minutes every day just putting in the same information ... when you are actually setting up the app on your phone is when you actually key that data in and then you don’t have to do it again

#### Compliance

The facilitators explained to the participants that the home-based completion of the chatbot would require dedicating approximately 40 minutes to the learning component, followed by a subsequent delay of 20‐30 minutes and another 10 minutes to complete the task. While participants expressed comfort with the overall duration of the task, the true challenge was quarantining the time to do this task.

I think anything more than 40 minutes it may be burdensome. I do think 40 minutes is the top. The challenge is disruptions and to really quarantine the time and be strong about that.

Participants proposed a solution, emphasizing the necessity of proactively earmarking dedicated time in advance to ensure the completion of the task. Having a large time window during which participants could complete the task would provide the flexibility needed for accommodating various schedules and preferences of participants.

I would quarantine in the time then for the next day and prepare myself because I don’t have any other commitments or pressures on my time.

Alternatively, participants suggested introducing a pause feature to mitigate any potential disruptions caused by unforeseen events, such as incoming phone calls or unexpected visitors. However, it is crucial to acknowledge that the introduction of a pause feature could potentially influence the integrity of the test. While these features may be convenient for the participants, this will introduce confounds to the results. Subsequently, a pause button was not implemented in Aurora.

I always find a pause button is important. For example: if I am starting the memory test or memory exercise right now and someone knock the door ... I have the opportunity that I can come back to that when I need to do something.

Another challenge identified was participants occasionally forgetting to complete the task. The facilitators explained to the participants that there are existing notifications within the app to remind the participants to do the task in the evening and in the morning. It was suggested that increasing the frequency of notifications could be a viable solution. Moreover, participants highlighted a tendency to ignore push notifications, particularly in the evening. To address this, participants proposed implementing alternative reminder methods, such as phone calls or text messages. Participants identified that their interest and motivation to complete the memory task were closely tied to the app’s clarity. The consensus was that instructions and information presented in an easily digestible manner would keep participants’ engagement. It was evident that it is important to prioritize clear and straightforward instructions that would lead to sustained participant engagement, especially when participants are asked to complete the task on multiple occasions.

Okay, if you wanted me to use it repeatedly then it’s got to be clear and simple and easy to access if you’re talking about once every three months and that’s ok I’ll come back anytime.

Participants nearly unanimously highlighted the critical role of feedback on their task performance. They underscored the importance of receiving real-time feedback to gauge their progress and enable a sense of accomplishment and motivation to improve and continue to complete the task for multiple assessments.

I don’t want money but I do want feedback. I am involved in a lot of medical research I am surprised I don’t find out about the results.

Even participants who did not think that feedback was necessary thought it would be a welcomed supplementary feature, as it would motivate them to continue using the app. Subsequently, Sleep Memories integrated informative feedback to enhance user engagement and promote continued motivation. For others, the motivation was intrinsic (ie, feedback not needed), and people would remain motivated to contribute to science. Participants trusted that scientists knew what they were doing and would follow their guidance because they are experts in the field.

... The work that this unit does is so important. Just thanking them for their time and delivering, any material or rewards I find it degrading. I just want to donate my data to be used for the greater good. There is nothing you can give me to make it better.

## Discussion

### Principal Findings

This study aimed to investigate the perspectives of older adults both with subjective or objective cognitive impairment regarding barriers and facilitators to using an at-home assessment of SDM through a novel app named Sleep Memories. Through co-design methodologies, we assessed the initial prototype of the mobile app to incorporate user feedback into a revised version suitable for clinical testing.

We found that digitalization and technological advancements can both facilitate and hinder everyday life for older adults. This phenomenon is reflected in the term Janus-faced technology [29]. According to this concept, the successful integration of technology can substantially improve daily activities for older adults. However, barriers to use can evoke feelings of alienation and disconnection from the digital world. Understanding these barriers is essential to minimize these negative feelings and to, instead, develop accessible and user-friendly technology solutions for older adults, particularly those with cognitive impairments.

One of the key findings of our study was the importance of app flexibility in accommodating users’ diverse needs and preferences and in challenging barriers. While some individuals reported requiring high-level assistance, others indicated that they could navigate the app independently with minimal support. A recent systematic review highlighted the critical importance of tailoring technology to meet the specific needs and preferences of older adults with MCI and dementia [30]. Below we examine each of the facilitators and barriers we identified and explore corresponding, flexible solutions in light of existing research.

### Comparison to Prior Work

The “first night effect” is a confounding variable in sleep research where participants’ sleep is disrupted due to being in an unfamiliar environment. This effect has been studied in prior studies. For instance, one study [31] demonstrated its potential to impact sleep disorder diagnoses. The benefit of home-based testing is that participants can sleep in their usual environment, removing the need for multiple night sleep laboratory testing. Furthermore, the SDM data collected are not confounded by the first night effect and more accurately represent a participant’s true abilities than traditional SDM testing.

Within the discussion of voice responses, our findings align with prior work, with concerns from older adults regarding the accuracy of audio (eg, the chatbot’s ability to accurately capture verbal responses) and text (eg, spelling mistakes) inputs [32]. In response to these concerns, Sleep Memories has implemented a dual-layered validation process. First, an autocorrect function is used to rectify minor spelling inaccuracies automatically. Second, when the system is uncertain of the response, a request for confirmation by a researcher is made. The implementation and availability of a voice response option increase the accessibility of the mobile app for those who may have age-related declines or disabilities [32].

To increase the accessibility of mobile apps for older adults, it is important to navigate both technological and socioeconomic challenges. Our focus groups highlighted concerns like high bandwidth and data consumption, which could impede the functionality and availability of health apps such as “Sleep Memories” for older adults. Furthermore, older adults may have age-related physical limitations, such as arthritis or hand pain, which may impact their interaction with mobile devices. To address the high bandwidth and data consumption, we allow the app to be used without an internet connection, and data are uploaded when connectivity is available. As previously mentioned, the implementation of voice responses can be a solution to enable those with physical limitations to use the app.

Participants raised concerns about forgetting to complete the task, particularly for those who might overlook setting personal reminders. This aligns with previous research in the context of medication compliance, where it was observed that a single reminder was often insufficient to prompt action, suggesting that a backup notification could serve as a viable solution [33]. A solution in our context that was proposed was to increase the frequency and elevate the distinctiveness of reminder notifications (eg, for Sleep Memories, we implemented a unique auditory notification, specifically a vocal prompt stating “it is now time to complete your memory task”).

Similar to a previous study [34], we found that incorporating feedback mechanisms, like scores, within eHealth tools is imperative to foster user engagement and empowerment. These features motivate users by providing immediate, tangible feedback on their performance. However, it is essential for app developers and researchers to communicate the context and limitations of their scores, particularly when there is a lack of established normative data. Without proper context, participants may misinterpret their scores, leading to unnecessary anxiety or concerns.

Our study revealed that conducting tasks within a home setting may introduce various distractions. This was aligned with prior research on the environmental interference of unsupervised home cognitive assessments [26]. To mitigate this challenge, 2 solutions emerged during our focus group discussions. The first involves incentivizing participants’ engagement through targeted motivation strategies, such as feedback, to maintain focus despite potential distractions. The second suggests the need for flexibility in task design, allowing participants to pause and resume activities, accommodating the unpredictability of the home environment. However, this approach must be balanced against the risk of compromising data integrity. For instance, in “Sleep Memories,” participants can pause and take short breaks between trials without permitting interruptions during the trials themselves to safeguard data continuity and accuracy.

### Limitations

Our study comprised older adults who had relatively high mobile proficiency at least in the subdomains that we examined, so our findings may not be generalizable to older adults who have a low mobile proficiency. Another limitation is that our sample had less representation from individuals with MCI (n=3), which limited our ability to detect or uncover differences in responses related to cognitive impairment. Future research is necessary to explore this important area by recruiting a larger cohort of individuals with MCI. Similarly, our participants generally scored highly on the AMDPQ, meaning that they showed decent proficiency in mobile phone use. Whether these findings apply to older adults with low proficiency in mobile phones needs further investigation. Nonetheless, our study is one of the first to examine the potential barriers and facilitators to the use of mobile device apps, in particular to complete memory tasks, in those with cognitive impairment.

### Future Directions

In addition to the app modifications mentioned earlier, our findings provide valuable insights to meet the needs and preferences of our older adult users. When deploying Sleep Memories, we will first conduct a needs assessment to assess the ability to use existing and new technology as well as the user’s circumstances (eg, Do they have time to complete the task in the app without distraction?) and environment (eg, Do they have access to a technology-savvy person in their network?). Based on this assessment, we will offer users various levels of support such as the ability for a phone call by a researcher or various notification options.

### Conclusions

Our study demonstrates that while there are barriers related to accessibility, usability, and data integrity to using health-related phone apps in this population, there are important facilitators that can be implemented in phone apps to create a flexible and inclusive digital health tool. Furthermore, we have shown evidence on the potential feasibility of the “Sleep Memories” app to enable the collection of SDM data within a user’s natural environment.

## Supplementary material

10.2196/68147 Multimedia Appendix 1 Discussion guide for focus groups.

10.2196/68147 Multimedia Appendix 2 Chatbot for word pairs.

10.2196/68147 Multimedia Appendix 3 Abbreviated Mobile Device Proficiency Questionnaire.

## Abbreviations

AMDPQ: Abbreviated Mobile Device Proficiency Questionnaire
COREQ: Consolidated Criteria for Reporting Qualitative Research
HBA: Healthy Brain Ageing
MCI: mild cognitive impairment
SDM: sleep-dependent memory
