# Supplementary material for: Perceptions of the Use of Mobile Apps to Assess Sleep-Dependent Memory in Older Adults With Subjective and Objective Cognitive Impairment: Focus Group Approach
Source: JMIR Aging. 2025 Apr 28;8:e68147. doi: 10.2196/68147 (PMC12052296; doi:10.2196/68147)
Supplement: Multimedia Appendix 1 [file aging-v8-e68147-s001.docx]

| Welcome today and thank you all for coming today to participant in this focus group. My name is [researcher name] and I am one of the researchers at the Healthy Brain Ageing Clinic. Today’s focus group will run for about 90 minutes. We are currently looking at developing a mobile application that examines sleep-dependent memory. We are creating this new app for people like yourselves, who may need to have various types of tests to check their memory and thinking. However, before we develop the app, we want to seek the thoughts and opinion from our target users, to get some insight and make sure we are on the right track. So, we would like to lean on your expertise and get your assistance to help us develop the application.  We will spend the next hour by asking you about your experiences with mobile devices, interest in doing memory tasks through a mobile application. Then we will show you what we have planned for our mobile application, and we hope to get your feedback for this.  We need to do some housekeeping (*Slide 2*) before we set started. We welcome your feedback, so please be as open and constructive as you’d like. You can respond to as many or as few questions as you like. If you choose not to respond at all, that’s ok. You are free to leave the focus group at any time. There are no right or wrong answers.  As we have previously discussed, our conversations here today will be recorded using this audio recorder. We will review and transcribe the recording after this session and then destroy the recording. You will not be identifiable from this transcript as we will remove any names or identifying information. We now need your verbal consent that it is okay to start recording the session.  Here with me today is [second researcher name], who is also a member of the research team. Both of us will ask you some questions and will be taking notes as we go.  We will have a short break after 30 minutes.  Does anyone have questions before we get started?  **Introduction to SDM**  *Slide 3 – why are we interested in sleep-dependent memory?*  The type of memory we are interested in looking at is called ‘sleep-dependent memory’. This is the idea that newly learnt things during the day is rehearsed when we go to sleep. Sleep disruption, ageing, and cognitive decline can impair this function of sleep and form of memory. This is an area of sleep and memory that can be potentially targeted for treatment. However, there are barriers to studying sleep-dependent memory.  *Slide 4 – Why do we need to change how we study sleep-dependent memory?*  Sleep-dependent memory is usually completed in a sleep laboratory. Meaning you will have to stay overnight.   - You will have to sleep in a foreign environment   There is a need for someone trained to administer the task, which is not available for most sleep laboratories. We can only do small research studies because of the cost and difficulty to test this form of memory. As a result, we can’t integrate sleep-dependent memory into clinical trials. To improve sleep-dependent memory, we need to understand factors that affect it.  **Part 1: Initial Discussion (*Slide 5*)**  **Theme 1:**  **Current use of mobile phone apps for health and speech recognition**  **We would first like to hear about how you currently use your phone and your thoughts about completing the memory test at home on your own device.**   - Do you have a smartphone? What do you use it for? - What kind of apps do you currently use? - KEY: How many people use apps relating to their health? - Do any of you use any sleep-related apps? - What kinds of information (sleep-related) do these apps give you? - Do you find this information (sleep related) useful/ easy to understand?   - What do you like/ enjoy about using these apps?   - What do you dislike about these apps?   - KEY: Has anyone used speech functions on their mobiles (e.g., siri, alexa etc)? How do you find this? (reliability, ease of use, accuracy of understanding/communicating)   - How confident or trusting are you that the information provided is accurate?   **Part 2: Present the rest of the powerpoint slides**   - I would like to show you what we plan to develop in this research study. Please take a look at the screen. - We are proposing a mobile application that you can download from the app store or a link and complete at home on your own mobile phone. - The app will start by asking for your name, then it will ask you some demographic questions as well as the time you went to sleep. It will then go to sleep until it is ready for you to do the memory task. It will let you do the memory task three hours before your bedtime. - Here are the instructions for the memory task. *read instructions/give participants time to read the instructions*. - The words will appear on your mobile screen like this. - You will then be one word like this. You can then answer by text or by speech. - The task will take 40 minutes, then you can go to sleep. - When you wake up, it will ask you to confirm your sleep time and the number of hours your slept. - It will then ask you to complete the test again. - As well as a multiple choice section, which you can select the correct answer from four options.   **Theme 2: Interest in completing cognitive task through a mobile application**   - You have all experienced our Healthy Brain Ageing Clinic, where you completed a series of assessments with our clinicians looking at your memory and general health. What are your thoughts about completing components of the memory assessment via an app on your phone?   - KEY: Thoughts on practicality   - Thoughts on acceptability/level of comfort/confidence   - Thoughts on reliability of the technology   - Thoughts on what happens to the information (storage, transmission, access etc.) - If you were asked to complete a brief memory task through your phone, at home, instead of coming into a sleep laboratory, would you feel comfortable/willing?   - What might be some benefits of completing the assessment this way?   - KEY: What might be some challenges of completing the assessment this way?   **Theme 3: Chatbot, SDM task, and feedback**  **Part 3: Feedback for the mobile application**   - What are your general thoughts about this study? - Would you like to interact with a chatbot? Why/ Why not? - Do you think you might have any difficulties interacting with the chatbot? - Do you think that getting notifications and reminders would be beneficial? - Do you have any suggestions for improvement? How to make it more appealing/easy to use? - KEY: Do you think the alerts would be beneficial? - KEY: Do you think most people could complete the task alone without distraction for 40 minutes?   - What might be some strategies we could recommend to help with this?   - How long would you prefer the task to take?   **Task itself**   - Difficulty of the task? - KEY: Do you think it would be practical to do the task three hours before bedtime and within two hours of waking up? |
| --- |
