# Supplementary material for: Perceptions of the Use of Mobile Apps to Assess Sleep-Dependent Memory in Older Adults With Subjective and Objective Cognitive Impairment: Focus Group Approach
Source: JMIR Aging. 2025 Apr 28;8:e68147. doi: 10.2196/68147 (PMC12052296; doi:10.2196/68147)
Supplement: Multimedia Appendix 2 [file aging-v8-e68147-s002.docx]

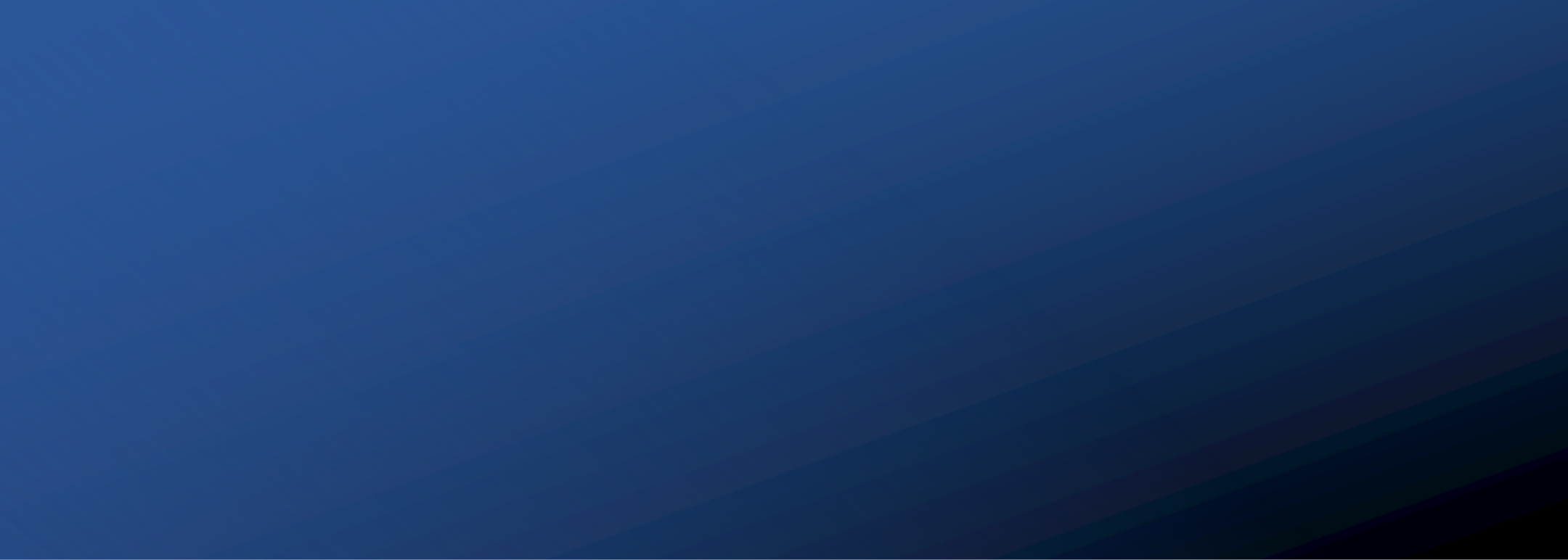

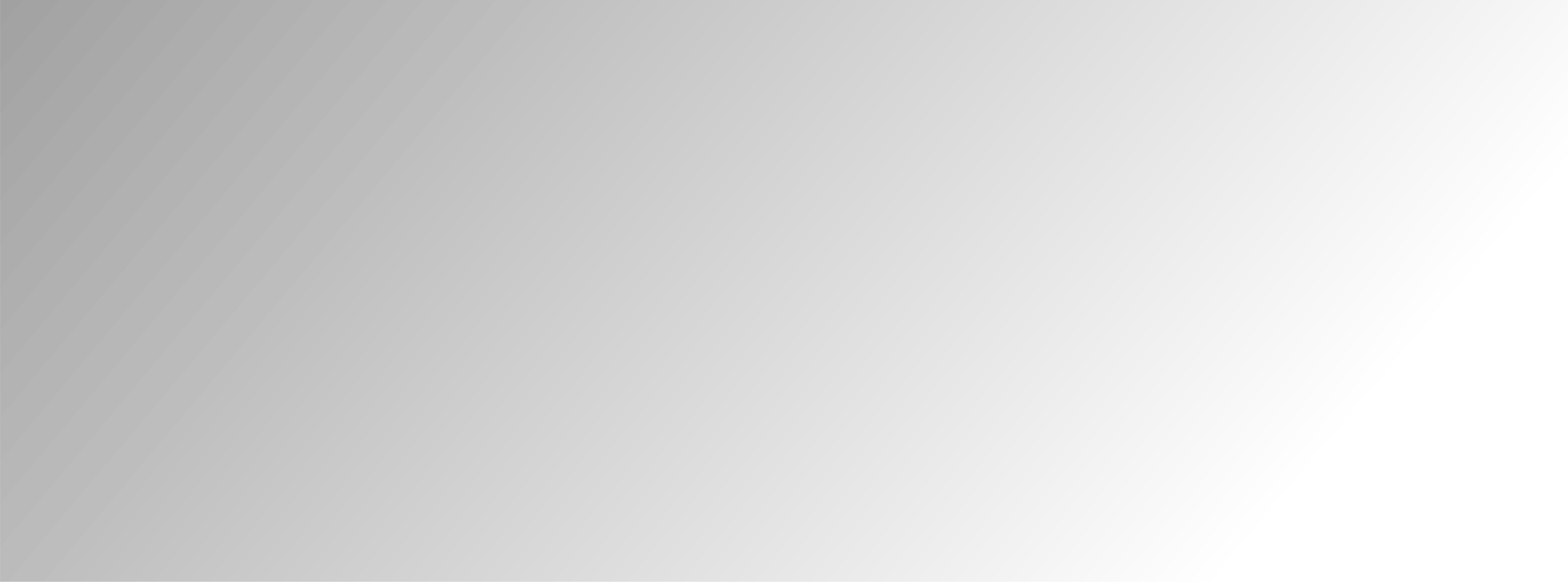

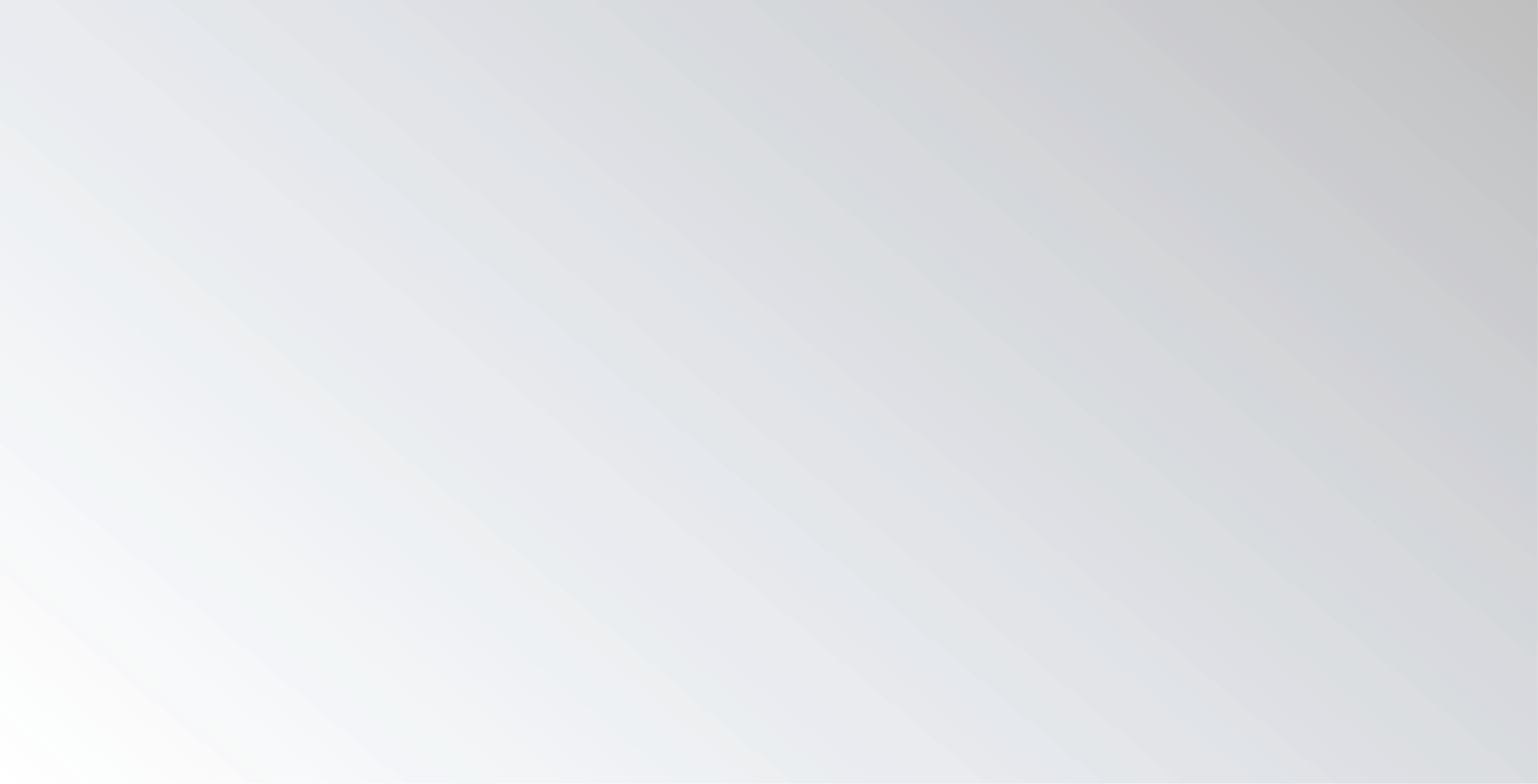

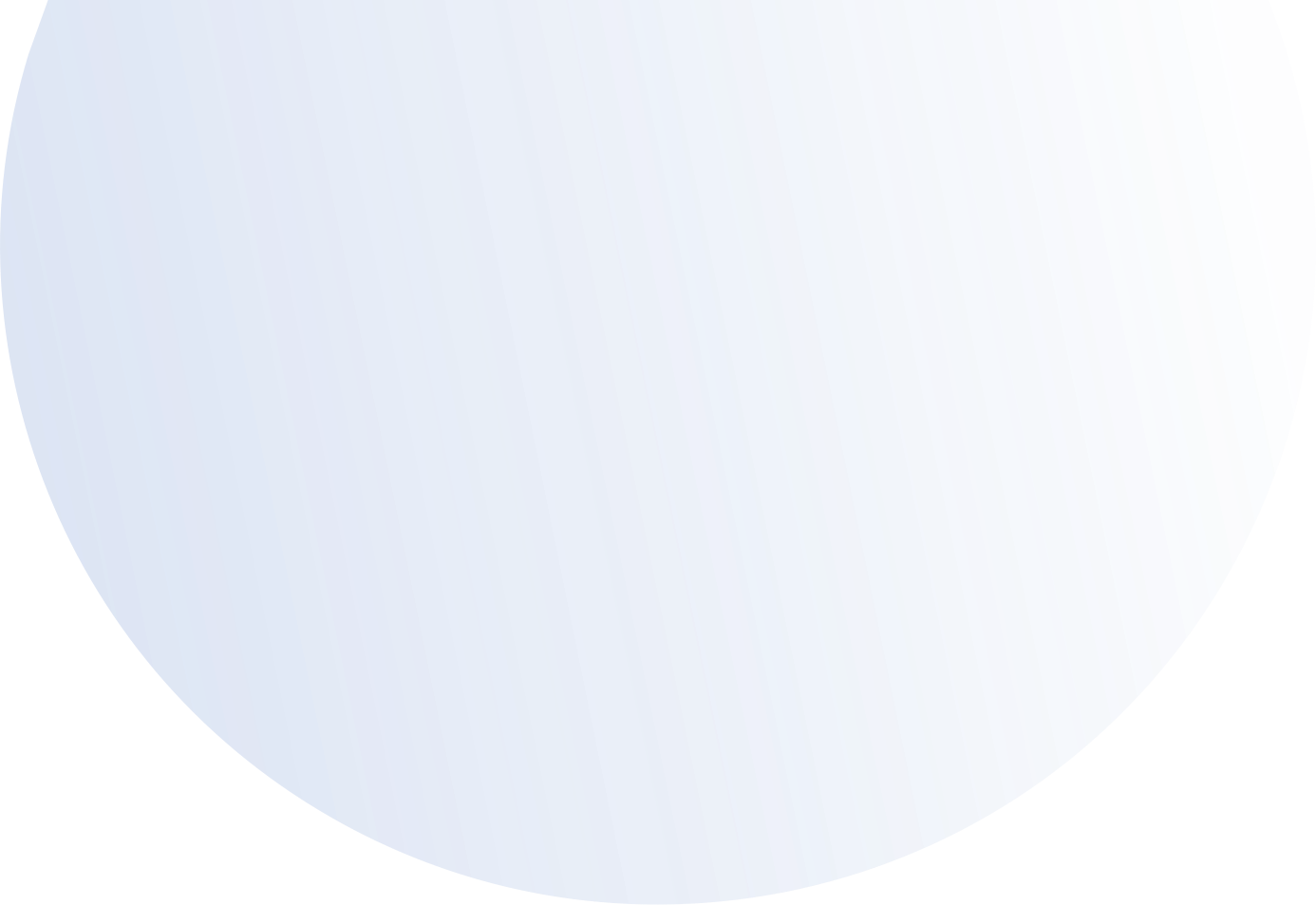


Chatbot for word pairs


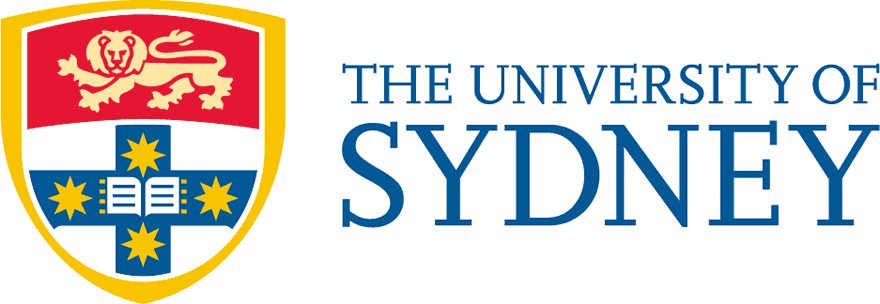


### Housekeeping


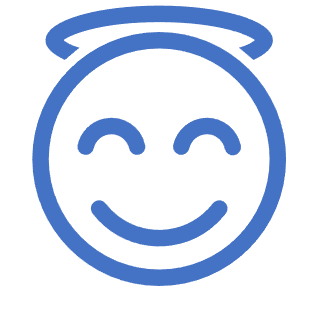


- Please be as open and constructive as you’d like.
- You can answer as many or as few questions as you like.
- There are no right or wrong answers.
- We will record the session.

# Why are we interested in sleep- dependent memory?

We know that things we learnt during the day is rehearsed when we go to sleep.

Previous work has shown that in ageing and cognitive decline this function of sleep can be impaired.

It is an area of sleep and memory that can potentially be targeted for treatment.

It is not commonly studied for various reasons, which we will go into later.

Why do we need to change how we study sleep-dependent memory?


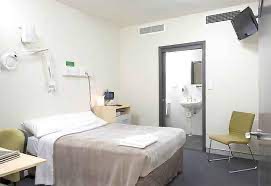


- Usually completed in a sleep laboratory
- Barriers:
  1. We need you to come stay in overnight
  2. Have someone trained to administer the task
  3. We can only do small studies
  4. We can’t integrate sleep- dependent memory testing into clinical trials.
- In order to improve memory, we need to understand factors that affect it.

#
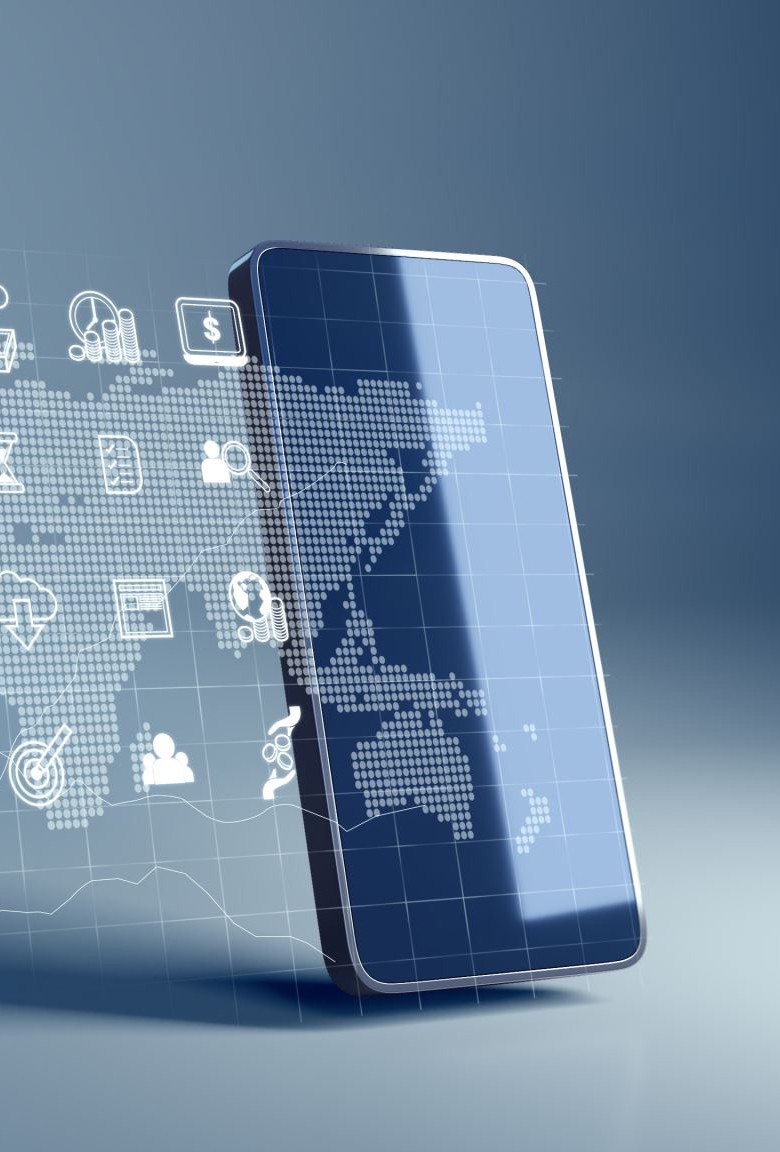
Theme 1: Current use of mobile phone apps for health and speech recognition

Tell us how you currently use your mobile phone for health apps.

- Any sleep-related apps?
- Do you use speech functions on your mobile phones (e.g., siri, alexa)

### What we are proposing


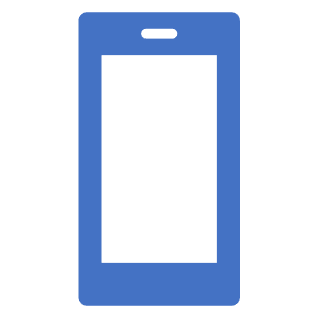


- - Having a mobile application that you can interact with and complete the task on your phone!

Potential benefits:

1. You can do it at home and have a normal night sleep after!
2. Don’t need to have trained staff present


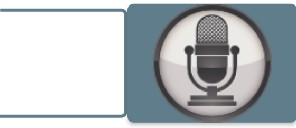


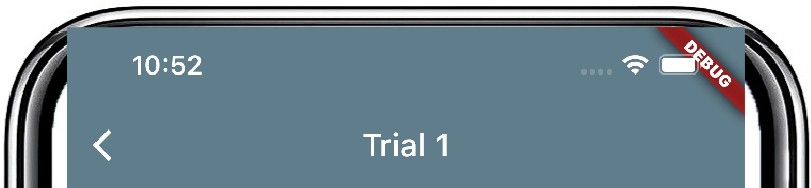


FORWARD

?


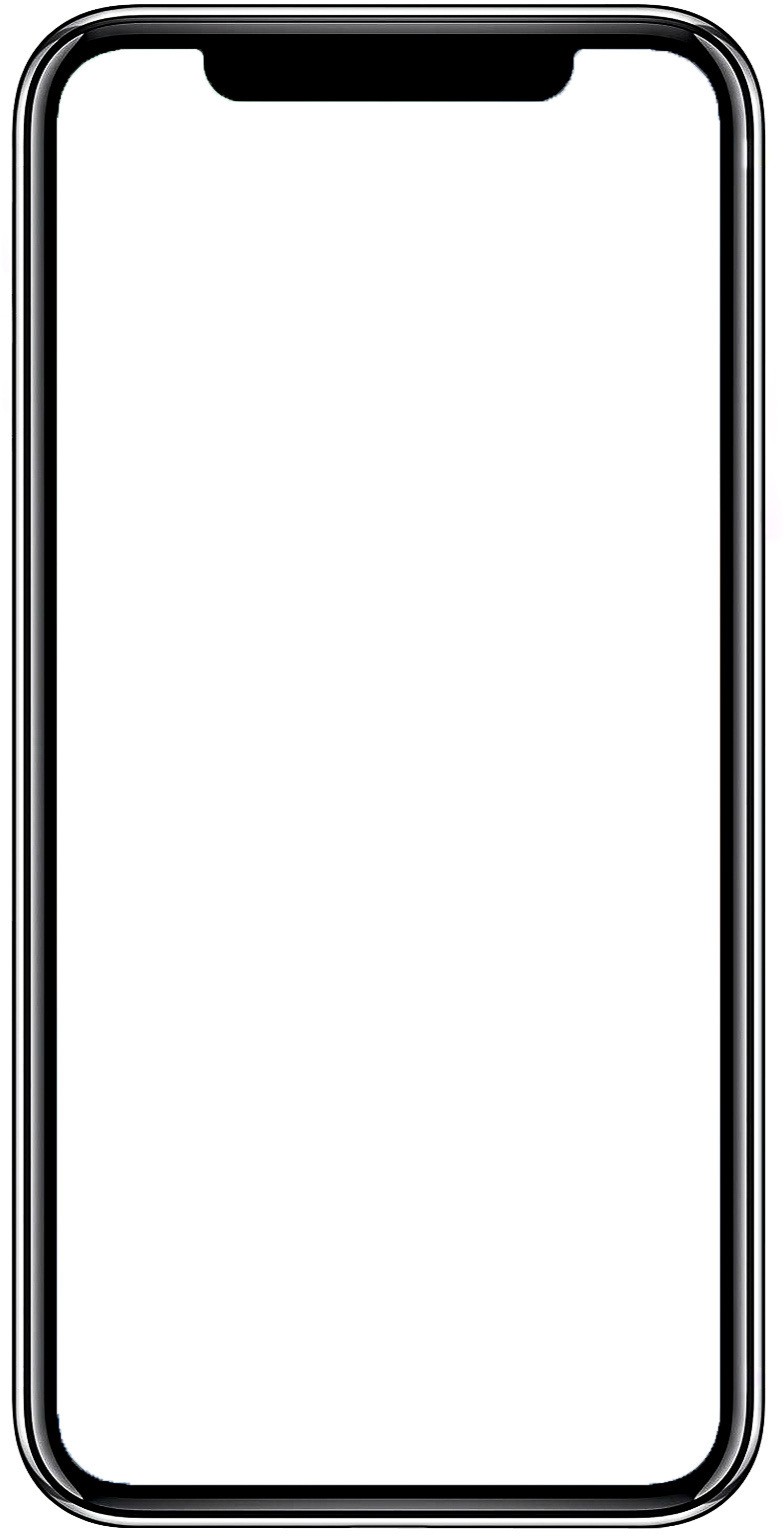

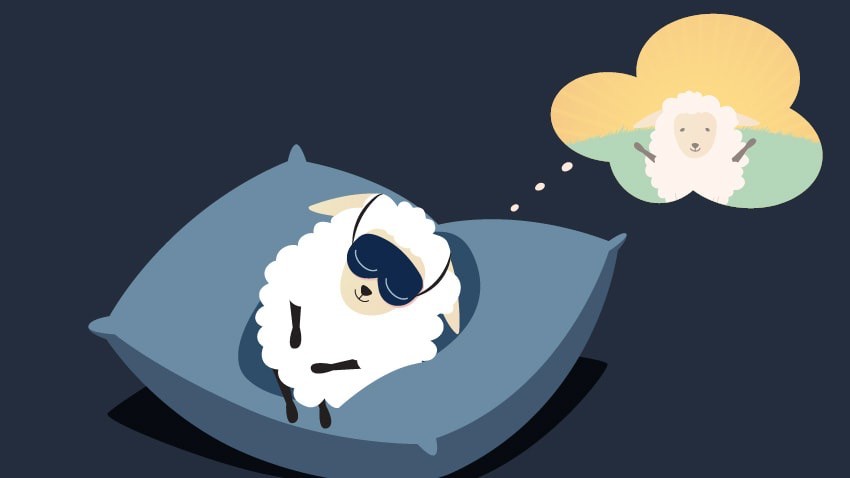

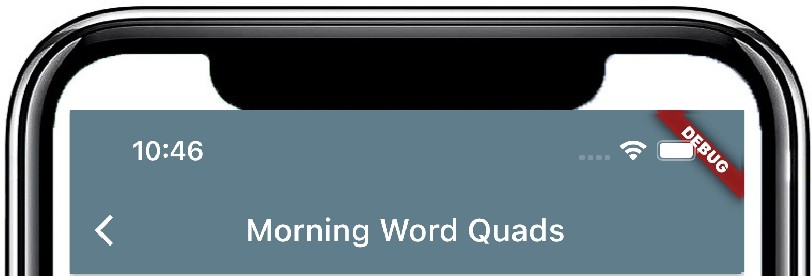


Far

Near

Lock

Close

Fright

## BREAK TIME!


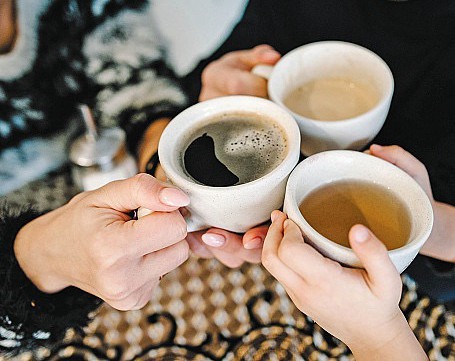

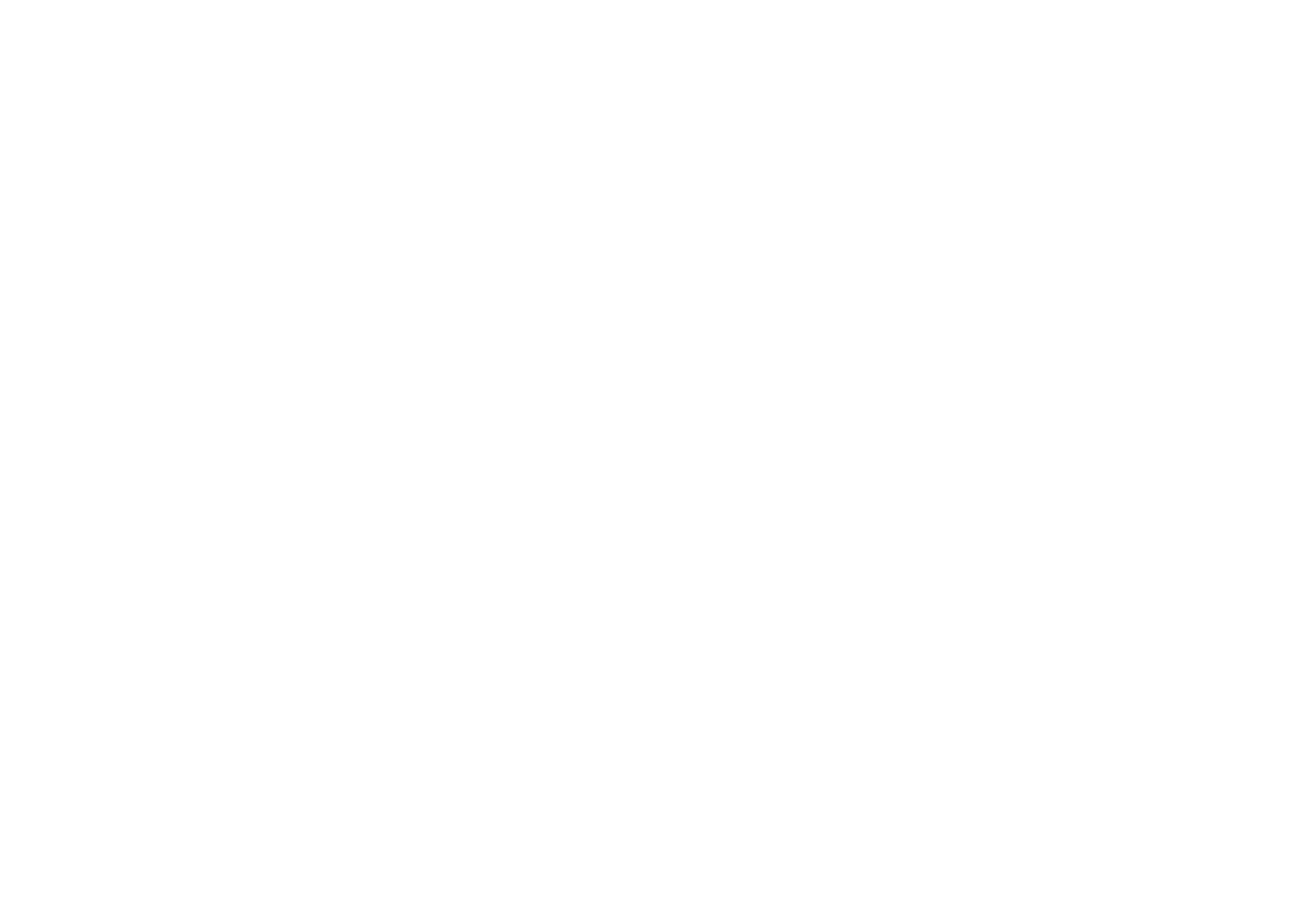


Theme 2: Interest in completing cognitive task through a mobile application


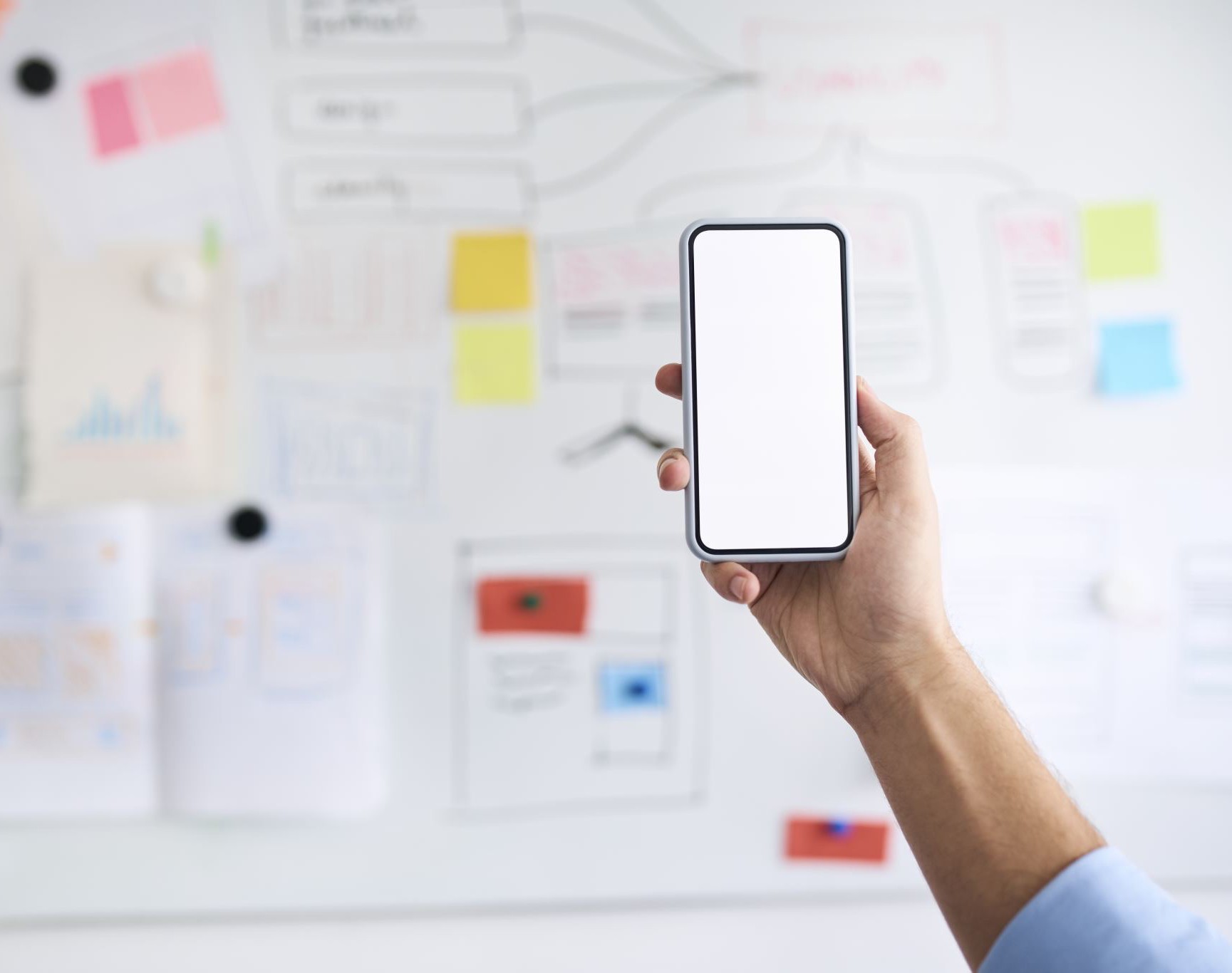

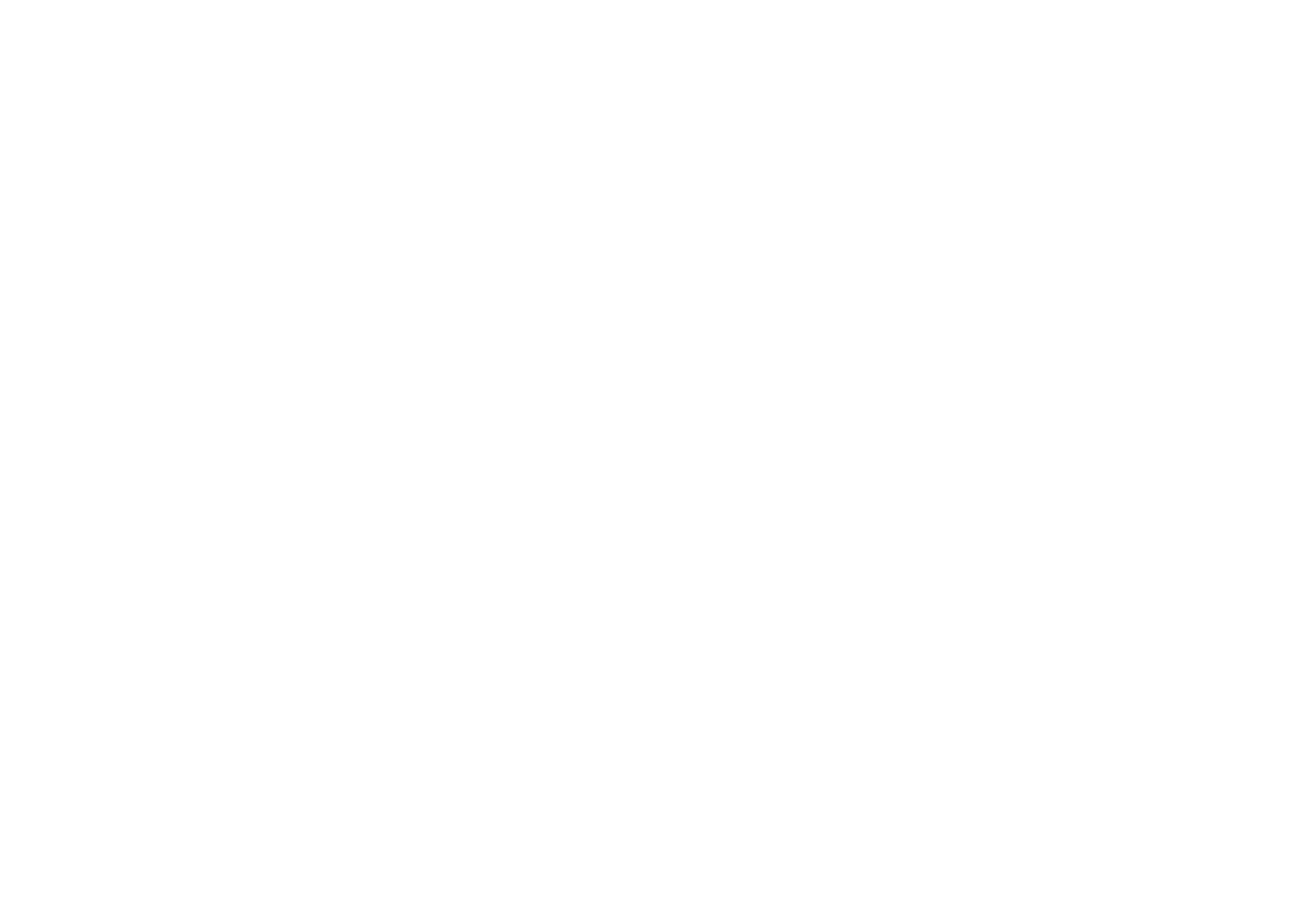

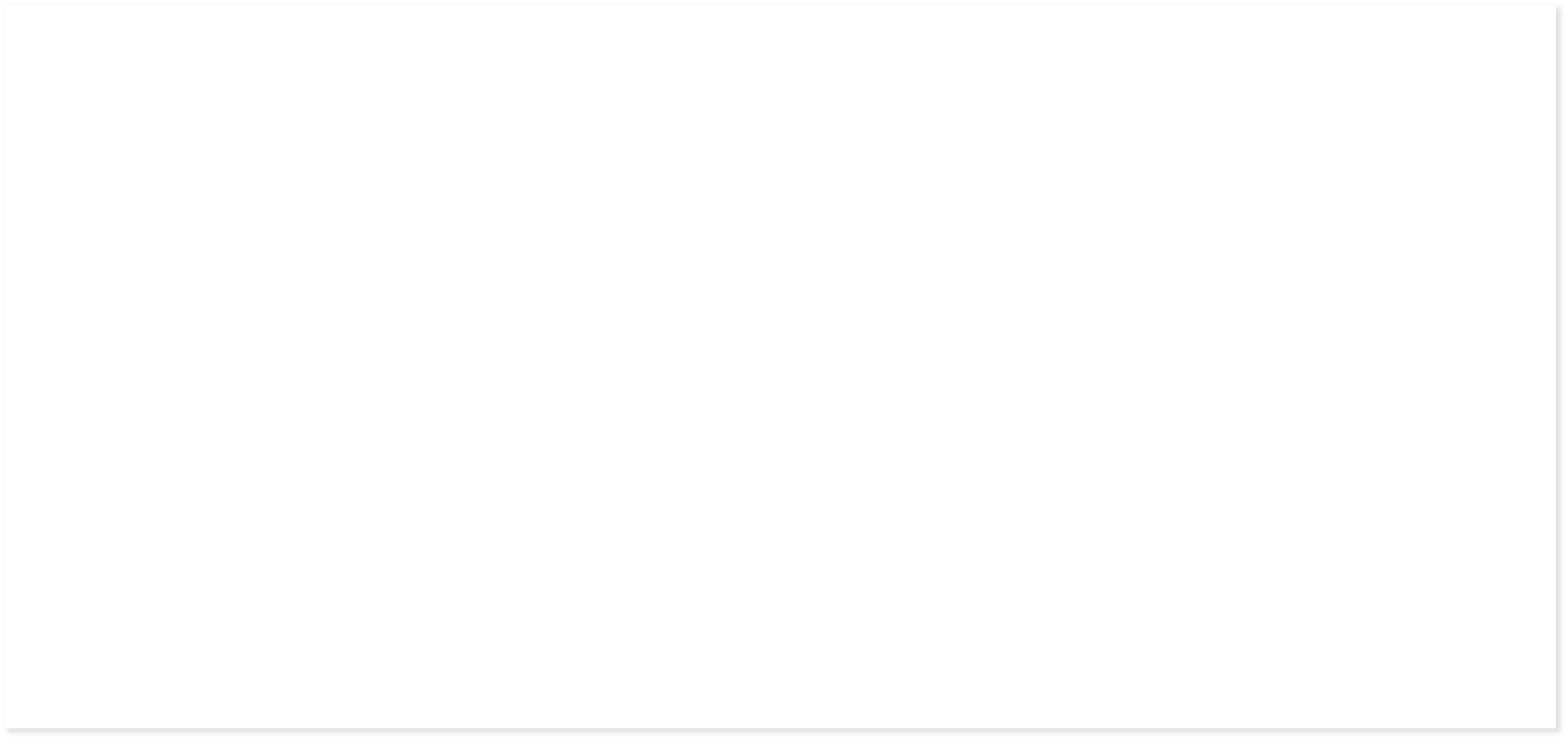


What are your thoughts about completing components of the memory assessment via an app on your phone?

- How practical is it for you?
- What might be some challenges of completing the assessment this way?

##
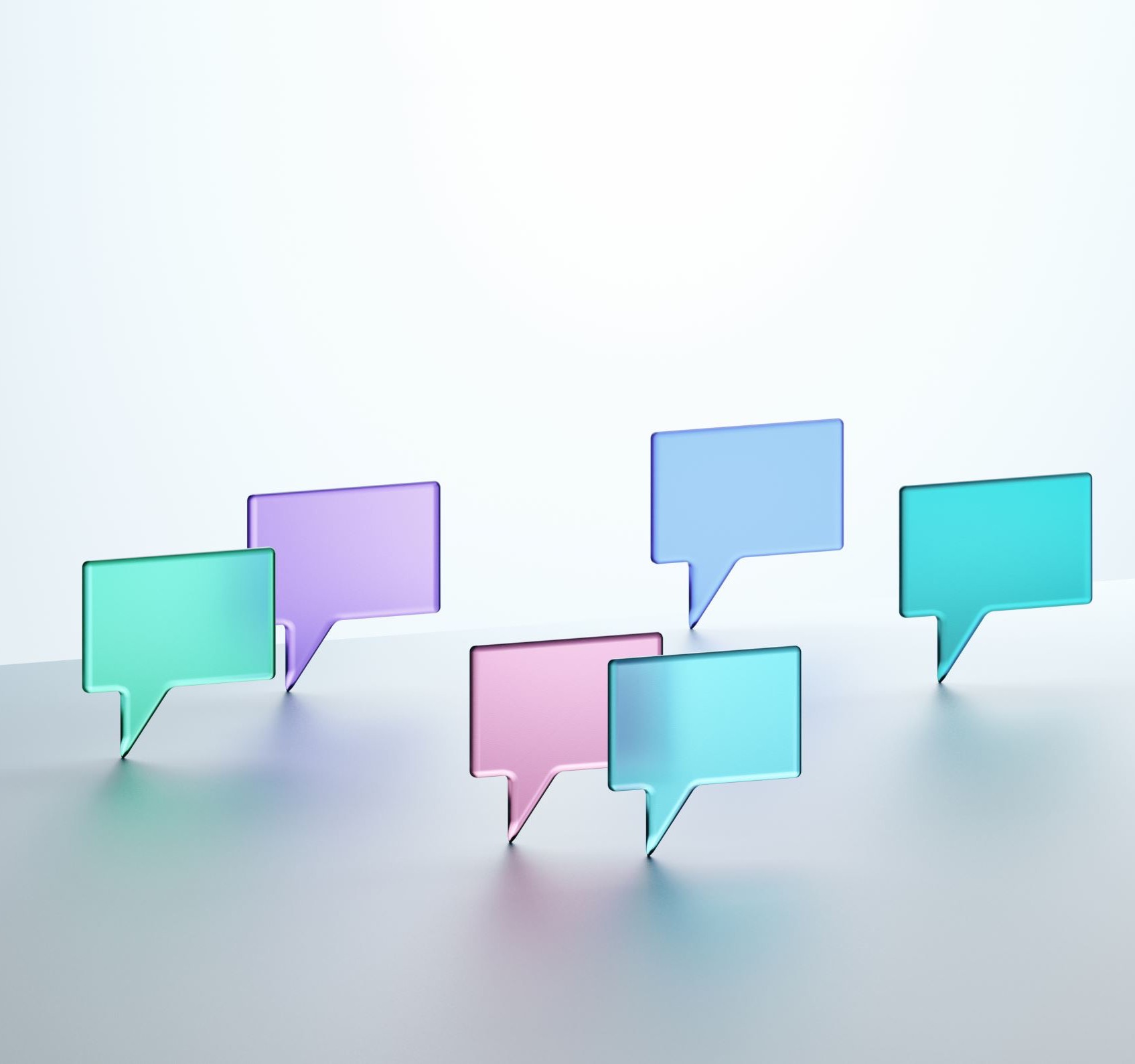
Theme 3: Chatbot, SDM task, and Feedback

### How does the mobile application look so far?

How can we improve it visually?


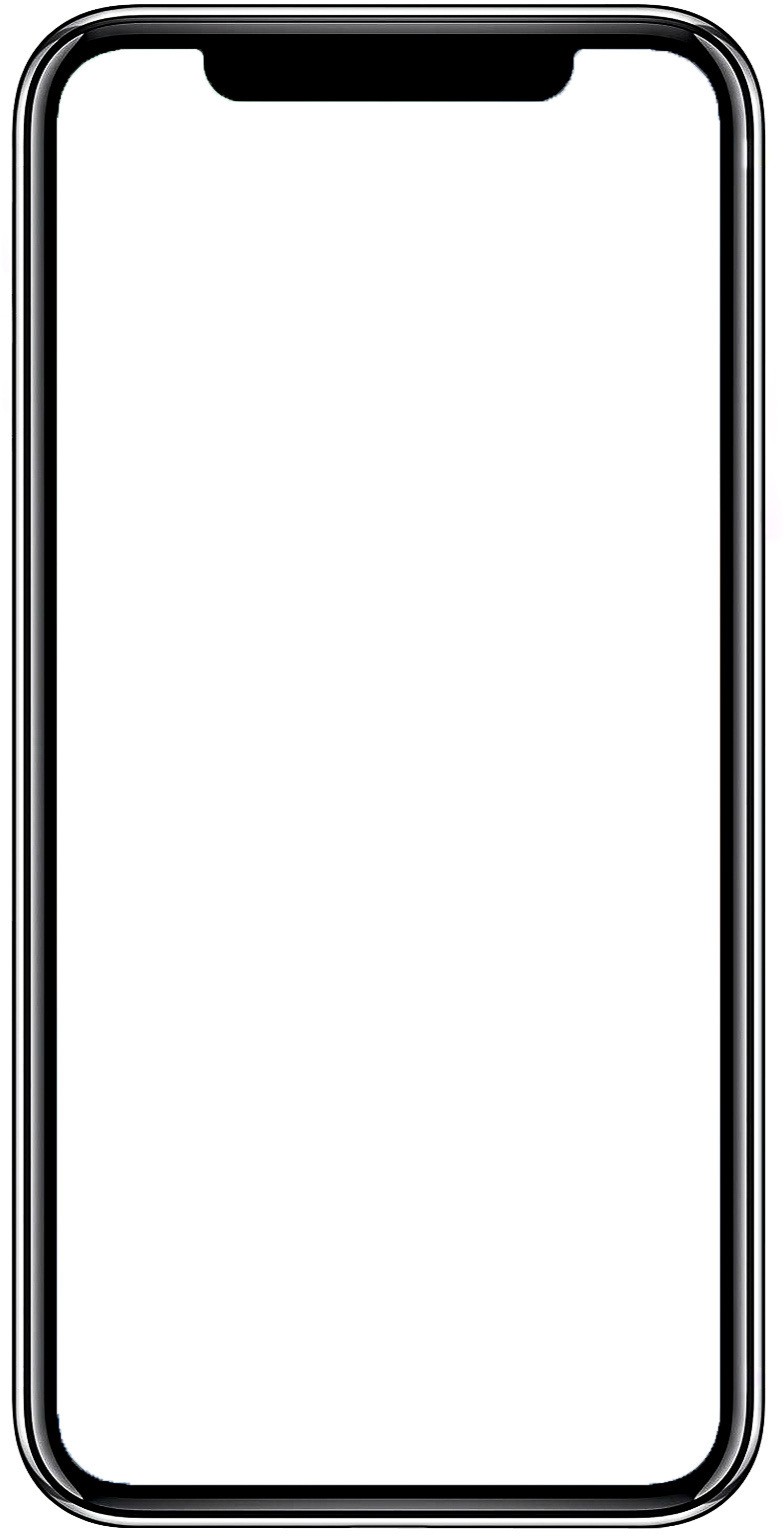

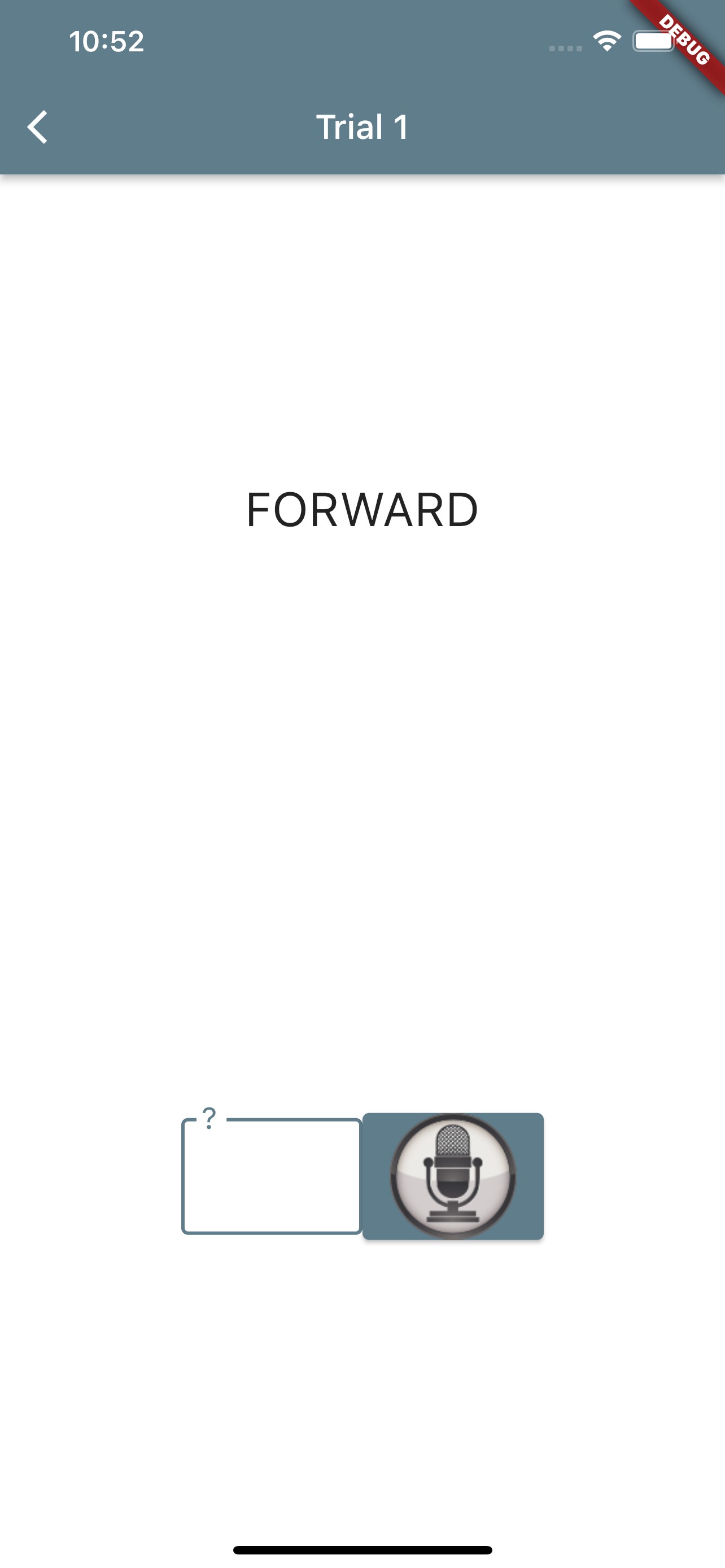


### Instructions

This memory task involves learning 32 word pairs and trying to remember them. This testing should take no more than 40 minutes.

You will be shown different sets of word pairs, on the screen. Some word pairs will make sense like bread and butter but others may not make sense like bike and sun.

### Instructions part 2

- I will show you 32 sets of word pairs like that. Please try to concentrate on the word pairs as they appear on the screen. After all the word pairs have been presented to you, I will show you the first word of each pair. And ask you to tell me which word went with it. Here is a practice:

### Practice

Movie

Apple

Orange

Prince

Many

Movie

Hands

Apple

?

Prince

?

?


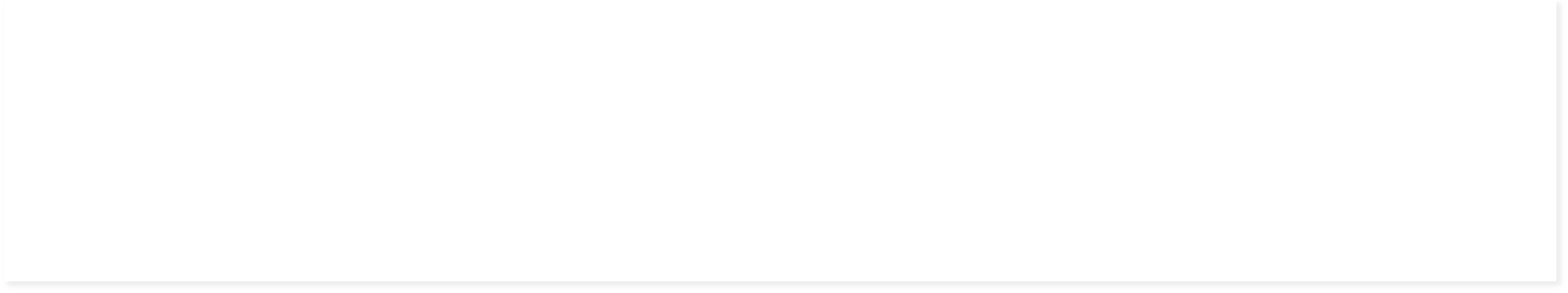


Feedback for the mobile application for far?

What might be some strategies we could recommend to help with encouraging you to complete it?


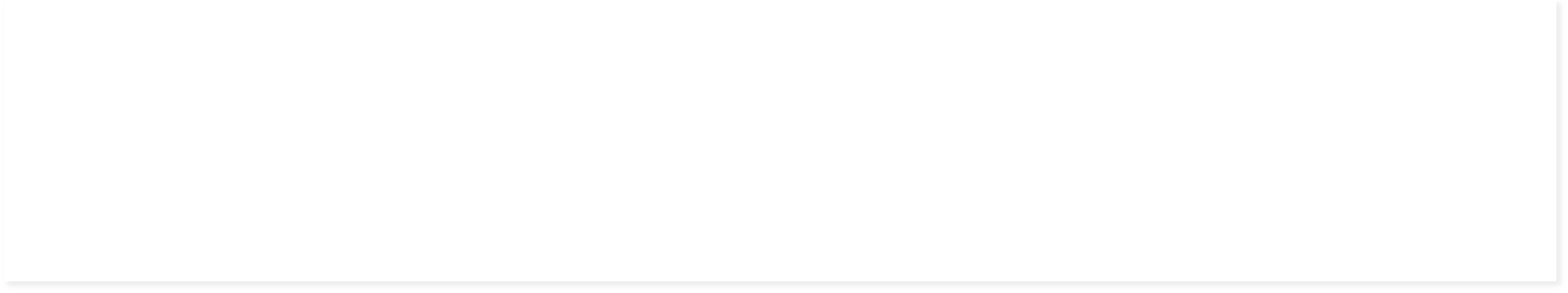


Any concerns about privacy?

- - We would like to collect your:
    - Name
    - Initials
    - Sex
    - Year of birth


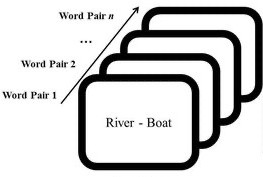


Word-pairs task

32 pairs of words

At night:

- - - Four learning trials

30-minute break

- - - One evening recall

Sleep

- - - Morning recall
