# Supplementary material for: Perceptions of the Use of Mobile Apps to Assess Sleep-Dependent Memory in Older Adults With Subjective and Objective Cognitive Impairment: Focus Group Approach
Source: JMIR Aging. 2025 Apr 28;8:e68147. doi: 10.2196/68147 (PMC12052296; doi:10.2196/68147)
Supplement: Multimedia Appendix 3 [file aging-v8-e68147-s003.docx]

|  | Never tried  (1) | Not at all  (2) | Not very easily  (3) | Somewhat easily  (4) | Very Easily  (5) |
| --- | --- | --- | --- | --- | --- |
| Mobile Device Basics | | | | | |
| Turn the device on and off | 0 | 0 | 0 | 1 | 9 |
| Charge the device when the battery is low | 0 | 0 | 0 | 0 | 10 |
| Navigate the onscreen menus using the touchscreen | 0 | 0 | 0 | 4 | 6 |
| Use the onscreen keyboard to type | 0 | 0 | 0 | 5 | 5 |
| Copy and paste text using the touchscreen | 1 | 2 | 1 | 2 | 4 |
| Adjust the volume of the device | 0 | 0 | 1 | 2 | 7 |
| Adjust the screen brightness | 2 | 1 | 0 | 2 | 5 |
| Communication | | | | | |
| Open emails | 1 | 0 | 0 | 0 | 9 |
| Send emails | 1 | 0 | 0 | 1 | 8 |
| Send the same email to multiple people | 1 | 1 | 1 | 1 | 6 |
| Store email address in an email address book or contact list | 1 | 1 | 2 | 1 | 5 |
| View pictures sent by email | 1 | 0 | 0 | 3 | 6 |
| Post messages to Social Media Networks (e.g., Facebook, Twitter, Instagram, Google Plus) | 2 | 0 | 1 | 4 | 3 |
| Use Instant-message (e.g., AIM, Yahoo Messenger, MSN Messenger) | 2 | 0 | 1 | 4 | 3 |
| Use video-messaging (e.g., Skype, Google Hangout, FaceTime) | 0 | 1 | 1 | 4 | 4 |
| Data and File Storage | | | | | |
| Transfer information (files such as music, pictures, documents) on my mobile device to my computer | 0 | 1 | 1 | 3 | 5 |
| Transfer information (files such as music, picture, documents) on my computer to my mobile device | 2 | 1 | 0 | 3 | 4 |
| Store information with a service that lets me view my files from anywhere (e.g., Dropbox, Google Drive, Microsoft Onedrive) | 2 | 1 | 1 | 3 | 3 |
| Internet | | | | | |
| Use search engines (e.g., Google, Bing) | 1 | 0 | 0 | 2 | 7 |
| Find information about local community resources on the internet | 0 | 0 | 0 | 3 | 7 |
| Find information about my hobbies and interests on the internet | 1 | 0 | 0 | 2 | 7 |
| Find health information on the internet | 1 | 0 | 0 | 2 | 7 |
| Read the news on the internet | 1 | 0 | 0 | 2 | 7 |
| Make purchases on the internet | 2 | 0 | 2 | 1 | 5 |
| Bookmark website to find them again later (make favourites) | 1 | 1 | 1 | 4 | 3 |
| Save text and images I find on the internet | 1 | 1 | 2 | 3 | 3 |
| Troubleshooting and Software Management | | | | | |
| Restart the device when it is frozen or not working right | 0 | 0 | 2 | 5 | 3 |
| Updating games and other applications | 1 | 1 | 0 | 3 | 4 |
| Close games and other applications | 1 | 1 | 0 | 1 | 7 |
| Delete games and other applications | 0 | 1 | 2 | 2 | 5 |
| Upgrade device software | 0 | 1 | 1 | 2 | 6 |
| The number of participants that selected each response is presented. Ten out of eleven participants completed the questionnaire. | | | | | |
